# Risk of African swine fever introduction into the European Union through transport-associated routes: returning trucks and waste from international ships and planes

**DOI:** 10.1186/1746-6148-8-149

**Published:** 2012-08-30

**Authors:** Lina Mur, Beatriz Martínez-López, José Manuel Sánchez-Vizcaíno

**Affiliations:** 1 VISAVET Center and Animal Health Department, Veterinary School, Complutense University of Madrid, Avenida Puerta de Hierro s/n, 28040, Madrid, Spain

**Keywords:** African swine fever, Risk assessment, Transport, Trucks, Waste disposal

## Abstract

**Background:**

The uncontrolled presence of African swine fever (ASF) in Russian Federation (RF) poses a serious risk to the whole European Union (EU) pig industry. Although trade of pigs and their products is banned since the official notification in June 2007, the potential introduction of ASF virus (ASFV) may occur by other routes, which are very frequent in ASF, and more difficult to control, such as contaminated waste or infected vehicles. This study was intended to estimate the risk of ASFV introduction into the EU through three types of transport routes: returning trucks, waste from international ships and waste from international planes, which will be referred here as transport-associated routes (TAR). Since no detailed and official information was available for these routes, a semi-quantitative model based on the weighted combination of risk factors was developed to estimate the risk of ASFV introduction by TAR. Relative weights for combination of different risk factors as well as validation of the model results were obtained by an expert opinion elicitation.

**Results:**

Model results indicate that the relative risk for ASFV introduction through TAR in most of the EU countries (16) is low, although some countries, specifically Poland and Lithuania, concentrate high levels of risk, the returning trucks route being the analyzed TAR that currently poses the highest risk for ASFV introduction into the EU. The spatial distribution of the risk of ASFV introduction varies importantly between the analyzed introduction routes. Results also highlight the need to increase the awareness and precautions for ASF prevention, particularly ensuring truck disinfection, to minimize the potential risk of entrance into the EU.

**Conclusions:**

This study presents the first assessment of ASF introduction into the EU through TAR. The innovative model developed here could be used in data scarce situations for estimating the relative risk associated to each EU country. This simple methodology provides a rapid and easy to interpret results on risk that may be used for a target and cost-effective allocation of resources to prevent disease introduction.

## Background

African swine fever (ASF) is one of the most devastating diseases of swine due to the high mortality caused (mainly in the hyper-acute and acute forms of the disease), the absence of effective vaccine, and the severe trade restrictions associated with its presence in the affected areas. ASF is caused by the infection of ASF virus (ASFV) which is transmitted by direct contact with fluids and excretions from infected animals. Blood, widely present in hyper-acute and acute forms of the disease, is considered the major route of direct transmission, containing high titers of virus for long lasting periods [1]. Other common ways of ASFV infection are the bites of infected ticks, and the indirect contact with various contaminated fomites and pig products, where ASFV remains infectious for long periods. As an example, ASFV infectivity persists more than 1000 days in frozen meat [2], 15 weeks in putrefied blood stored at room temperature [3] and one month in contaminated pig pens [4]. This long ASFV persistence explains that the introduction of contaminated pork meat or other pig products from international transports and, its eventual use to feed pigs, is one of the most frequents ways for ASFV introduction into free territories. For example, this route has been hypothesized to be the way of introduction in Europe, specifically in Portugal in 1957, in the Caribbean sea (Cuba) in 1971, in South America (Brazil) in 1978, Belgium in 1985 or, recently, in Georgia in 2007 [5].

Other fomites (e.g. vehicles, animal feed, veterinarians or contaminated material) have been also identified as important routes for pig diseases introduction/spread into free-territories [6]. In fact, returning trucks have been identified as one of the most important ways of spread of diseases such as classical swine fever (CSF) by studies performed in Denmark and The Netherlands [7,8]. Unfortunately, the risk associated to this route is not easy to be quantified, mainly because of the lack of detailed information and the need to estimate large number of parameters which increases the complexity and uncertainty of the models to be used. Some studies have assessed the introduction of some animal diseases through returning trucks in specific regions or countries [7,8] or through illegal meat [9]. Other were aimed to estimate the exposure of susceptible populations to swill or catering waste (e.g. [10-12]) in countries such as Denmark, The Netherlands, United States of America or United Kingdom. Even, recently published studies addressed the risk of ASFV introduction by imports of pigs [13]. However, to the best of the author´s knowledge, no studies have assessed the risk of ASFV introduction through transport-associated routes (TAR) in the whole European Union (EU).

After the introduction in Georgia in 2007, the spread of ASFV into Trans Caucasian countries (TCC) and Russian Federation (RF) territories has caused more than 200 notifications, with more than 120000 animals culled in the area [14]. Currently, ASFV spread is not controlled within RF, with continuous occurrence of outbreaks. Moreover, some of those outbreaks have been notified in very distant regions (>2500 km) from the initial outbreaks and are close (<150 km) to the EU borders [15]. In response to this situation, the EU authorities carried out some scientific studies to estimate the risk of ASFV introduction into the EU [16], which highlighted the need for the proper disinfection of returning livestock vehicles coming from affected areas [17].

Considering i) the severe sanitary and economic impact of ASF in the affected territories, ii) the current situation out of control in RF [15]; iii) the closeness of some ASFV outbreaks to the EU borders and iv) the evident concern of the EU authorities regarding the risk associated with returning trucks [17], it was considered valuable to estimate the potential risk of ASFV introduction into the EU by TAR. The semi-quantitative model presented here provides a simple and transparent method to interpret results, identifying the most critical TAR for each country and ranking the countries based on their relative risk for ASFV introduction.

## Methods

The study presented here was aimed to estimate what the World Organization for Animal Health (OIE) guidelines of risk analysis referred to as “release or entry assessment” [18] for the three TAR analyzed. As we have no information and detailed data regarding the subsequent infection associated with contaminated trucks and with the potential illegal use of waste for animal feeding purposes for each of the 27 EU countries, exposure assessment was not evaluated in this work.

In collaboration with the Veterinary Epidemiology and Public Health group from the Royal Veterinary College (RVC) a semi-quantitative risk assessment model was developed to estimate the risk of ASFV introduction by the selected routes, using the combination of principles from the knowledge driven spatial models [19] and expert opinion elicitation methods previously used in other animal health risk assessments [20,21]. Risk factors related with the risk of ASFV introduction by each route were selected based on literature review. Information for these risk factors was gathered and further combined weighting their importance by expert opinion (EO) to obtain the relative risk for each country by each route. Consequently, this study was structured in three consecutive steps: (1) risk pathways definition, (2) likelihood estimation, and, (3) sensitivity analysis.

### Risk pathways definition

The goal of the study was to estimate the spatial variation of the risk of ASFV introduction into the EU countries by TAR and to identify the TAR at highest risk for ASFV entrance in each specific country. Based on historical data, current awareness and previous works [16] about ASF risk of introduction into EU, three TAR were considered in the model. The first one was referred as returning trucks and was intended to measure the probability of ASFV introduction into the EU by potentially contaminated livestock vehicles (i.e.. trucks) coming from affected areas. The second TAR was the waste disposal from international ships, which historically, has been one of the most important ways for ASFV introduction into free areas. The third TAR was the waste from international planes.

### Likelihood estimation

An intensive literature review was performed to select and collect information about the risk factors that could be used to estimate the risk of ASFV introduction by each of the considered TAR pathways. Those risk factors, for which complete, reliable and updated data was available for all the EU countries, were selected for the model calculations. Sources of data, main assumptions and uncertainty associated to each of these risk factors have been summarized in Table 1.

**Table 1:** Description of the risk factors used in the model for ASFV introduction into EU by TAR

**Name**	**Risk factors**	**Parameter to be estimated**	**Data source**	**Assumptions**	**Uncertainty**
**P1**	Number of live pigs exported from EU to ASF-affected countries by road	Number of potential ASF contaminated returning trucks	[22] (Nov. 2007–2009)	Only pig exports to TCC and RF were considered.	Other type of trucks could also get in contact with ASFV in affected areas; however the most probable is that a pig truck enters into a farm.
				It was assumed that trucks that export live pigs may enter into a farm and potentially become contaminated with ASFV.	
**P2**	Number of the roads crossing EU national boundaries with non-EU states	Number of ways (and consequently, facility) of a truck to arrive by road to an EU country from non-EU countries.	[23]	Borders with all non-EU member states were included except members of the European Free Trade Association (Switzerland, Lichtenstein, Norway).	Other factors such as cultural relations, effectiveness and quality of controls or topography, were not considered.
				It was assumed that higher number of cross border points, implies higher number of connections, and consequently easier to share trucks movements.	
**P3**	Three scenarios were used to approximate the proportion of returning trucks not properly disinfected	Returning trucks not properly disinfected	[7]	Despite disinfection of returning trucks from ASF affected areas is mandatory, this measure is not always 100% effective.	As no field data is available related with efficiency of this measure, common scenarios were used for the 27 EU countries. If known, differences between countries may be simulated within the model by the selection of different scenarios in each country.
				For the best scenario, a 5% of returning trucks not properly disinfected was assumed, 15% for the medium scenario, and 25% for the worst case scenario.	
**P4**	Inward number of cargo ships from ASF-infected countries to EU ports	Potential ASF-contaminated waste introduced by cargo ships	[22]; [14]	Imports of goods were considered without differences between products. More volume of goods implies more waste.	Catering used in the cargo ship not necessary comes from the departure country.
**P5**	Inward number of passenger ships coming from ASF-infected countries to EU	Potential ASF-contaminated waste introduced by passenger ships (excluding cruises)	[22]; [14]	More passengers imply more catering and consequently, more waste.	Passenger ships not always have catering and do not imply that food comes from origin countries.
**P6**	Short sea shipping (SSS) ships coming from ASF-infected countries to EU	Potential ASF-contaminated waste introduced by SSS movements	[22]; GIS	Volume of goods transported by SSS movements by Baltic and Black sea. Only two sea regions were considered as potential risk for ASF introduction (Baltic and Black sea).	Higher volume of transported products not always implies higher number of crew on the boat and consequently higher volume of catering and food brought from origin countries.
**P7**	Proportion of cruise ships coming from ASF-affected areas by country	Potential ASF-contaminated waste introduced by cruises	$P7=\frac{CA_{i}}{\left[C_{p}/p\right]}$	Assuming that these cruises bring catering food from departure or call countries.	A potential stop in an affected country does not always imply use of food from this country.
					Unknown origin of cruise catering increase uncertainty of this measure.
**CA**		Number of cruise ships arriving at EU ports after one stop in ASF-infected areas.	[24]	Assuming a similar number of cruises and origins in the different years.	Data from one year to another may change
**Cp**		Number of cruise passengers arriving at EU ports (Cp)	[22]		Data from one year to another may change
**P**		Average number of passenger by cruise ship was used to estimate number of cruisers (P)	[25]	Assuming a similar number of passengers by cruise.	Different types of cruises with different capacities could affect the final estimation
**P8**	Commercial passenger flights from ASF-infected countries to EU airports	Potential contaminated waste introduced by international passenger flights	[22];[14]	It was assumed that commercial flights from affected areas could potentially bring food from origin countries. The higher the number of flights from ASF-infected countries, the higher the risk of using ASF contaminated products.	Unknown origin of the catering increase uncertainty of this measure.

Detailed data about assumptions, uncertainties and data source of each risk factor used in the model was included in the table.

The risk associated with returning trucks was estimated using three risk factors which were: (1) number of pig exports toASF-affected countries (i.e RF and TCC) (2) number of road border cross points with non EU countries (except borders with Switzerland, Lichtenstein and Norway) and (3) proportion of returning trucks not being properly disinfected. This last risk factor was defined using three different scenarios that allow the evaluation of the effect of preventive measures (i.e. high, medium and low percentage of truck disinfection) on the final risk values. For final calculations, medium scenario proportion of truck not properly disinfected was selected for all the countries.

In order to estimate the risk associated with waste disposal from international ships, we used the volume of goods and number of persons transported by different types of ships as an indicator of the volume of the potential contaminated products arriving by ships to the EU. For the purpose here, only movements coming from ASF-infected countries were considered for the analysis. Particularly, four different types of ship movements were considered, based on EUROSTAT official classification. The first type is cargo ships, which includes all type of ships for transporting goods. The second type is passenger ships, which includes ferries and other boats used for human transportation, excluding cruises. Short sea shipping (SSS) is the third type of ships, which includes movements of goods within small seas. Specifically here, only movements in Baltic and Black sea were considered, as they are surrounded by ASF infected countries. The fourth and final type of boat is cruises, particularly, only cruisers with at least one call in an ASF-infected country (African countries and RF) were considered in the analysis.

Similar to boat waste estimation, the number of commercial flights coming from ASF-affected areas was selected as risk factor for the volume of potential ASF-contaminated waste arriving by plane to the EU.

### Risk factor standardization

Country data for each risk factor was obtained and further standardized into the same comparable scale to allow their comparison and further combination. Specifically, for each risk factor, data values of all the EU countries were transformed into six categories based on country data distribution. These categories are based on Natural Breaks of the data countries values for the specific risk factor, further adjusted using the Jenks optimization method of ArcGIS 9.3 (ESRI) [26]. For each RF, countries values were transformed into values from 0 to 5. For each category, a risk level was assigned, considering the following ranking: negligible (0); very low (>0 - ≤1); low (>1- ≤2); medium (>2 - ≤3); high (>3 - ≤4); very high (>4).

### Expert opinion elicitation

Final results for each TAR were obtained by the weighted combination of the risk factor categories. To estimate the importance and consequent weight for each risk factor and route, an EO panel session was performed in Lisbon, September 23^rd^ 2011, during the ASFRISK symposium. EO elicitation process have been widely used in many fields (engineering, sciences, social or medical research among others) to deal with uncertainties and gather information on parameters not formally described in literature. These methods are based on the opinions or judgments of experts in the specific field to be covered [27]. In this case, the group of experts included twenty-three international experts, with wide experience on animal health risk management, including three CVOs from EU countries, eight representatives from EU CVOs, and representatives from FAO, OIE, DG SANCO and Russian Agriculture Ministry, and highly experienced researchers in ASF.

The selected methodology for developing the EO sessions was a Delphi modified method, similarly to those used by Gale et al. and Gallagher et al. in previous works. Following basic steps of Delphi method, first of all, experts were given an introduction about the goal of the risk assessment, the structure and risk factors of the model, as well as the instructions for voting. Each expert was given an electronic remote control device associated with an interactive presentation. This system guarantees the anonymity of the votes and enables the on time review of the votes, allowing the discussion and comments about them on the same session.

During the EO session, experts answered ten closed questions with a number of possible answers, for the comparison of the importance of each risk factor by route and within routes. The results of the EO were combined assigning equal weights to all the experts, which allows to obtain the weights by RF and route. These weights were computed using the percentage of votes that considered each RF/route as the highest risk for ASFV introduction.

### Combining the results

Once all the weights were obtained by EO and each risk factor value was standardized, both were combined in order to obtain the relative risk of ASF introduction into the EU by TAR (RR_TAR_). The way to combine these risk factor values (numerical values) was a linear weighted model, similar to those used in the knowledge driven models [19], using the weights obtained during the EO elicitation. The detailed explanation of this combination is described in the following formulas:

(1) $$\text{RRTAR}=\sum_{\text{i}=1}^{\text{n}}[0\text{.65(R.T})+0\text{.24(W.S})+0\text{.11(W.P})]$$

$\text{RRTAR}=\sum_{\text{i}=1}^{\text{n}}\left[0.65\;\left(0\text{.33P1i}+0\text{.33P2i}+0\text{.33P3i}\right)+0.24\;\left(0\text{.33P4i}+0\text{.42P5i}+0\text{.24P6i}+0\text{.01P7i}\right)+0\text{.11P8i}\right]$ where *n* is the number of countries evaluated for ASF-TAR risk in the EU, *R.T.* is the risk value for returning trucks route and, *WS* and *WP* are the risk values for waste disposal from ships and planes, respectively. The second formula disaggregates the routes by risk factors used for its calculations, which definitions, data sources and uncertainties are explained in Table 1.

The final risk values were translated into qualitative results, following the same risk categories explained before, to facilitate the comprehension of the results.

### Sensitivity analysis

**Figure 1:**
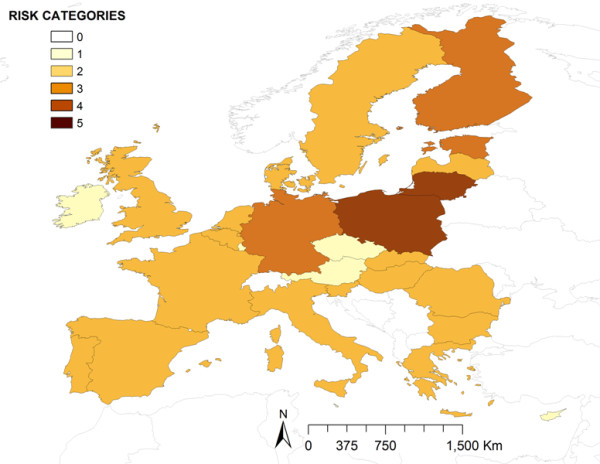
Distribution of the relative risk for ASFV introduction into the EU by transport-associated routes.

All weights used in the model were analyzed in the sensitivity analysis (SA) by the 25% increase and decrease from their initial values, considering twenty different SA scenarios. Results obtained in each of these twenty SA scenarios were compared with the results obtained in the reference model (Figure 1). Correlation between results of these scenarios and the reference results was calculated by using the Spearman correlation coefficient (Rho) calculated with R-language (v. 2.14.1, R Foundation for Statistical Computing, Vienna, 2010). Additionally, the number of countries changing their risk category in the SA scenarios was represented.

All results were represented using choropleth maps with ArcGIS 9.3.1 (ESRI).

## Results

Most of the EU countries (16 over 27) presented a low level of risk for ASFV introduction by TAR (RR_TAR_). The minimum risk (0.53) was located in Malta and the maximum risk (3.52) in Poland. The countries at highest risk were Poland and Lithuania followed by Finland, Estonia and Germany, with medium risk (Figure 1).

**Figure 2:**
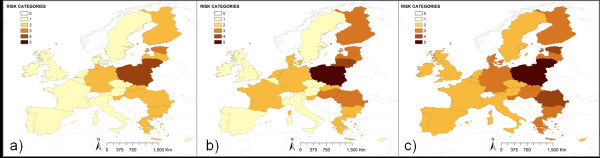
**Relative risk of ASFV introduction by returning trucks when considering three different scenarios.** Three different scenarios were used to approximate the proportion number of returning trucks not properly disinfected **a**) 5% (best scenario), **b**) 15% (medium scenario) and **c**) 25% (worst scenario).

The highest risk for ASF introduction was associated with returning trucks, accounting for the 65% of the total TAR risk. In the scenario with medium proportion of disinfected trucks, Poland had an estimated very high risk (Figure. 2) followed by Lithuania with high risk, and Estonia, Finland, Latvia and Romania, with medium risk, being all of them neighboring countries to current affected area of RF. As it is shown in Figure 2, the level of disinfection of trucks meaningfully changes risk categories, from level two (low risk) in the case of Romania for the best-case scenario, to level four (high risk) in the worst-case scenario.

**Figure 3:**
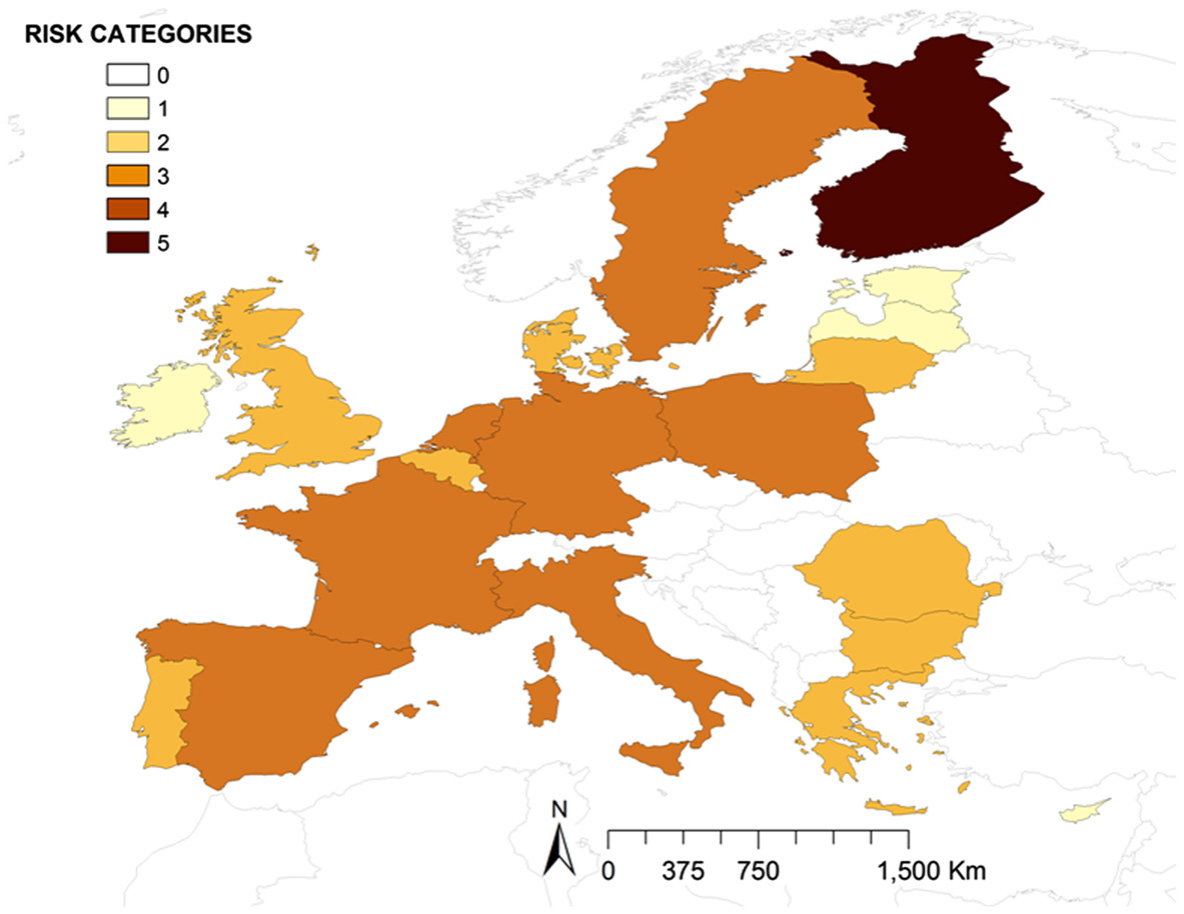
Relative risk of ASFV introduction into EU by waste from international ships.

**Figure 4:**
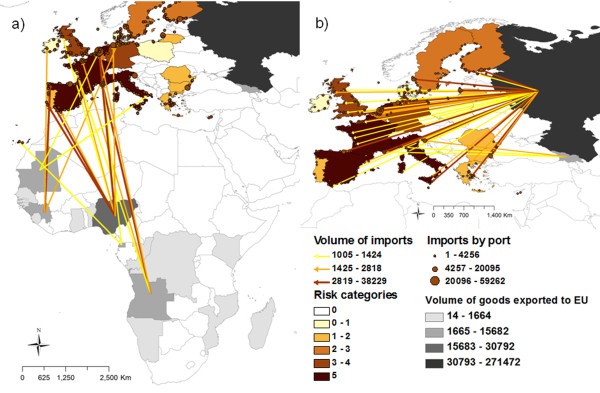
**Relative risk of ASFV introduction into EU by waste from cargo ships.** Results for the relative risk of ASFV introduction into EU by waste from cargo ships are represented with detail of the origin and destination of imports coming from Africa ( **a**) and European ASF-infected countries ( **b**).

Waste from international ships was the second most relevant pathway in terms of risk. Risk map for this pathway (Figure 3) highlighted Finland with a very high risk, followed by several countries with a medium risk. Nevertheless, some countries concentrated very high risk of ASFV introduction associated to specific types of ships. For movements of passengers, considered as the most important type of ship movements by EO, only Finland and Poland registered movements from ASF affected areas, concentrating a very high and medium risk, respectively. In the case of cargo ships, the second most important boat type, four countries concentrated a very high level of risk (Figure 4). The detailed analysis of this type of movements revealed that the main origin of these cargo ships movements was RF, followed by Nigeria and Angola, however different patterns of risk were found among destination countries. For SSS movements, four countries surrounding the Baltic sea (Denmark, Finland, Lithuania and Sweden) as well as Bulgaria in the Black sea, concentrated very high levels of risk. For cruise movements only five North European countries concentrated very high risk levels, although the relative importance of this type of movements was considered low by EO.

**Figure 5:**
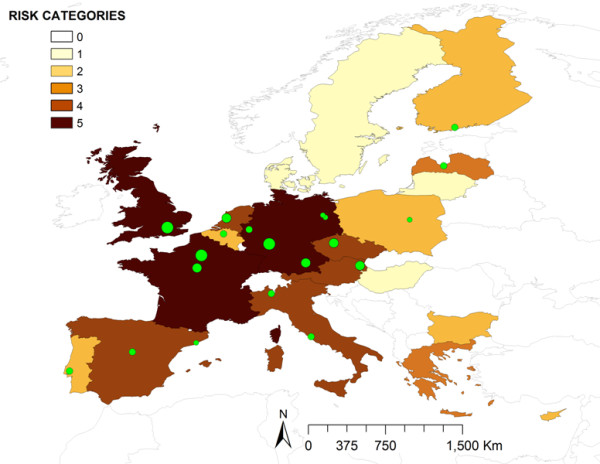
**Relative risk of ASFV introduction into EU by waste from international flights.** EU airports are shown in green dots graduated by the volume of extra-EU flights coming from ASF affected areas.

Finally, waste from international planes was the pathway posing highest risk for United Kingdom, France and Germany (Figure 5). The main EU airports are represented in Figure 5 using graduated symbols based on the number of extra-EU commercial flights received from ASF-affected countries.

**Figure 6:**
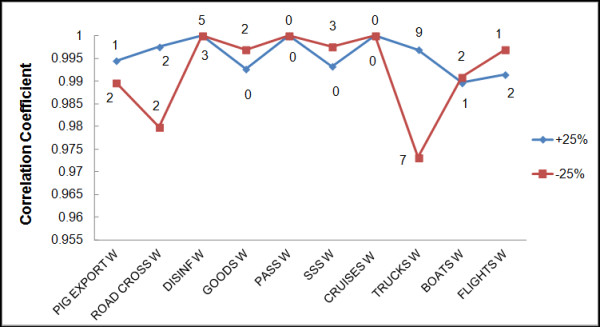
**Correlation between sensitivity analysis scenarios changing risk factor weights (+ − 25%) and the reference model results.** The number of countries that changed their risk category in the different scenarios is represented near the line.

SA revealed that the model is robust. The lowest correlation coefficient obtained between the reference scenario and each of the different SA scenarios was Rho = 0.97 (*p* < 0.01) (Figure 6). Analyzing the impact that changes in inputs had on the country risk category, none of the countries experienced a change greater than one category. All these findings revealed that substantial changes of 25% in the initial weight values do not meaningfully affect final risk results.

## Discussion

This is the first study aimed to assess the risk of ASFV introduction into the EU associated to TAR. Current presence of the disease without control in areas of RF close to the EU borders, together with results of the EFSA advise [16], other studies [28] and recent published legislation [17], point out the importance that illegal trade and other potential pathways such as transport fomites may have in the ASFV introduction into the EU, which remarks the importance of the study here.

However, the estimation of TAR is not a simple task. Vehicles and waste from international means of transport have been frequently suggested as a potential route for disease spread, specifically for ASFV introduction into free areas [29,30]; but no studies have quantified this risk. The lack of information for these TAR does not allow to use traditional risk assessment models making necessary to develop alternative approaches to analyze the risk of ASFV introduction by these pathways. The methodology proposed here combines methods used in the knowledge driven models used for spatial modeling of diseases [19] as well as methods for the risk estimation based on expert opinion elicitation [20,21]. Moreover, we used available data on risk factors, conveniently standardized, weighted by EO and linearly combined (as done in spatial modeling), to estimate the relative risk of TAR in the different EU countries. Although results should be cautiously interpreted considering all the assumptions and uncertainties associated with the model structure and data used, the approach is believed to be useful to evaluate the TAR risk. This study was specifically performed considering the selected routes of entrance, and, importantly, the specific characteristics of the pathogen, for the risk tended to be estimated. For example, the long survival of ASFV in all kind of meat and infected products allows to measure the risk based on potential incoming volumes of infected material, without considering the survival time of the virus on it. However, when adapting the methodology presented here for other animal diseases or routes of entrance, this important feature, as well as many other specific characteristics and parameters, should be modified conveniently to incorporate the features of the disease under study.

One of the most important aspects to be considered is that the model does not provide probabilities, but compares the relative risk between the 27 EU countries based on the risk factors evaluated. Indeed, a high value on the model results does not imply an absolute high level of risk, but a higher one compared with other EU members. On the other hand, the selection of information for each of the risk factors used in the model is influenced by the quality and availability of data sources for the 27 EU countries. For example, the degree of cleaning of returning trucks is based on scenario rather than real data due to the absence of this data for each of the EU countries. Therefore the results presented here depend on the quality and reliability of this data. It is important to consider also that the model only estimates the risk of entrance/release of potential ASFV-contaminated material/transports, but does not consider the subsequent exposure of the susceptible livestock population in the destination country.

Another idiosyncrasy of the model is the use of weights obtained by EO for combination of risk factor and pathways. EO process is a valuable method widely use when no other “more objective” information is available (i.e. literature, etc.) and particularly for the estimation of complex parameters or parameters with significant uncertainties, as some of the presented in this study. Particularly, Delphi approach is one of the most frequently used methods of EO that originally does not allow for interaction between the experts [27]. However, in this study small modifications were made by the little interaction between experts during the results presentation and the use of electronic devices for voting. This technique implies many advantages, being an adequate way to collect information for solving problems. However, the lack of universal guidelines or standardized procedures for its performance could arrive into difficulties that should be cautiously considered [31]. Some problems of the technique could be derived from the inappropriate selection of the experts, the lack of previous information, the inadequate performance of the questionnaire or the combination of the results. Nevertheless, this method may provide a more realistic and updated view of the scenarios under evaluation, in this case related with ASF risk, based on the experts valuable experience. Moreover, and because weights used in the weighted combination of the risk factor are a critical aspect of the model, an intensive SA was performed in order to identify the impact of these estimated weights in the final results. This SA reveals that the model is robust and do not significantly change when changing the weights provided by EO. In fact, none of the countries changed more than one category in the different SA scenarios evaluated. For example, the 25% decrease on the weight of returning trucks, which is the scenario with lowest correlation coefficient (Rho = 0.97), affected categories of seven countries. Most of them (three countries) changed from low to very low risk, two decreased from medium to low risk, and two from high to medium risk. These changes result in a very similar risk map, but with a slight difference of risk category in these countries, which confirms the robustness of the model.

At the same time, the use of different scenarios in some of the measured parameters allows Animal Health (AH) Authorities in each EU country to have the possibility to select the scenario that more realistically represents their current situation based on their expert opinion. For example, we are providing three different results based on certain assumptions, but AH Authorities may consider that for their countries only the scenario one is realistic, so they will have the possibility to select it and visualize the correspondent outputs. This flexibility, as well as, the easy update and the possibility of incorporation of more detailed information (if available for some countries) instead of being considered a limitation, is considered as one of the main strengths of the model.

Model results reveal that the median of the risk values for ASFV by TAR in the 27 EU countries is low (for 16 of the 27 EU countries), although big differences were found between countries and pathways. An expected result of the model is that EU countries closer to RF and TCC borders are the ones at higher risk for ASFV introduction by TAR, being Lithuania and Poland the countries at higher risk for ASFV entrance, followed by Finland, Estonia and Germany. Returning trucks is the TAR at highest risk for ASFV introduction into the UE, being almost three and six times more important than waste from ships and planes, respectively. This result is in agreement with the EU commission risk perception which recently approved a legislation [17] that strengthen and remind the importance of cleaning and disinfection for returning livestock vehicles coming from affected areas. In fact, the differences found in the results of the three scenarios for the disinfection of trucks (Figure 2) highlight the importance of that measure in preventing the entrance of animal diseases into free territories. On the other hand, ships waste has two times higher risk than plane waste. Ships waste has been recently suggested as the origin of the outbreaks in the Caucasus region [5], which may have influenced the opinion of the experts regarding their weights. Again we should highlight that the risk associated to ships and planes waste would depend not only on the release in the EU country, but on the final exposure, or contact, with susceptible populations, and this fact has not been measured on this work.

The analysis in detail of the results obtained for the different countries and the different analyzed pathways give us a better characterization of the risk. For example, in the case of Lithuania, although it has an overall high risk of ASF introduction by TAR, this risk is mostly associated to trucks, but not to waste from international ships or planes. These results are certainly influenced by the geographic location of the country, close to the current affected area, and the intense commercial relations with RF, which has been demonstrated by the amount of pig exports to this country. The opposite case is Germany that resulted in a medium risk for the overall TAR, but only a very low level for returning trucks. In this case the presence of most of the EU airports (50%, five over ten) that receive large number of flights from ASF-affected countries [22] determines the high level of risk associated to waste from planes. Similarly, Germany is a very important country in maritime trade (cargo ships, SSS and cruises) which explains also the high risk associated to waste from ships. Other countries with high risk associated to waste from planes are France and United Kingdom, where the two most important airports in terms of number of flights coming from ASF-affected countries (Charles de Gaulle and Heathrow, respectively) are located (Figure 5).

Another interesting result is related with the big differences found among the different ship types. Although Finland is the unique country that concentrates an average very high risk for waste from ships pathway, other countries are only highlighted when a specific type of ship movements is analyzed. For example, Bulgaria has an estimated very high risk by SSS movements through Black sea, particularly associated with the port of Burgas, the second most important port in the Black sea [32]. Several countries surrounding the Baltic sea (Denmark, Finland, Lithuania or Sweden among others) are also highlighted in SSS movements and cruises, mainly due to their geographical closeness and trade with RF.

However, the most interesting results are related with waste from cargo ships, for which, four countries concentrate very high risk (France, Italy, the Netherlands and Spain). The detailed analysis of these movements, considering origin and EU destination ports, reveals some interesting differences among these countries. For example, in Netherlands, the risk both from RF as well as from Africa (mainly from Angola and Nigeria) is particularly concentrated in the port of Rotterdam, the one that receives the biggest amount of potentially risky cargo ships (those coming from ASF-affected countries) in the EU. However, in Spain the risk is more distributed, with two important ports receiving high number of cargo ships from Africa and other two from RF. In this particular case, these countries with the same level of risk present different profiles with one or several important ports in terms of risk. This fact enhances the importance of the detailed analysis of these results (represented in Figures 2, 3, 4 and 5) that could be much more informative to the policy makers than the general overview of the results (Figure 1).

Authors believe that this model has an important logical and biological approach as its results reflect areas and pathways identified at high risk by experts. This kind of models built using a simple and easy to understand methodology, are faster to develop and easier to interpret compared with the quantitative ones, and are particularly suitable when few information is available. For this reason this model may be considered as an adequate alternative in data scarce situations to provide a scientific support to risk managers, and ultimately, to prevent animal diseases introduction in free territories.

## Conclusions

We developed a semi-quantitative new risk assessment approach to estimate the relative risk for ASFV into each EU country associated with TAR. The absence of available data for this pathway and the complexity of the estimations due to the huge uncertainties associated, lead us to develop a semi-quantitative model based on the use of risk factors as risk estimators. The results of the model indicate that the median risk of ASF introduction in EU countries is low and mainly associated to returning trucks, although some countries concentrate higher levels of risk such as Poland and Lithuania. Methods and results of this study may help to allocate surveillance and other risk reduction measures to prevent or minimize the potential impact of ASF introduction into the EU.

## Competing interests

The authors declare that they have no competing interests.

## Author’s contribution

LM, BML and JMSV designed the model structure, the expert opinion session and decided the methodology to be used in the study. LM performed the data collection and model development. BML and JMSV critically reviewed the model results and suggested changes and adaptations to improve the quality of the study. All authors participated in drafting the manuscript, and have read and approved the final manuscript.
